# Assessing sexual practices and beliefs among university students in Khartoum, Sudan; a qualitative study

**DOI:** 10.1080/17290376.2021.2011390

**Published:** 2021-12-06

**Authors:** Husameddin Farouk Elshiekh, Hein de Vries, Ciska Hoving

**Affiliations:** Department of Health Promotion, School for Public Health and Primary Care (CAPHRI), Maastricht University, Maastricht, The Netherlands

**Keywords:** Sudan, HIV, sexual behaviours, university students, I-Change Model

## Abstract

University students in Sudan are more at risk of contracting HIV than the general population, due to a high rate of sexual activity and low uptake of preventive measures such as condoms. Hence, they are considered an important target for HIV prevention programmes. This study explored students` beliefs about abstinence and pre-marital sex. Thirty semi-structured individual interviews were conducted, based on constructs from the Integrated Change (I-Change) Model. The study sample included 16 (53%) male and 14 (47%) female university students. Their average age was 21.2 years (Range 18–27 and SD 2.5). Both sexual abstainers (*N* = 19) and sexually active students (*N* = 11) perceived HIV severity and susceptibility. Most of the participants had a positive attitude towards abstinence. However, sexually active students also perceived some advantages of engaging in sexual practices, such as sexual pleasure and proving adulthood. Sexually active students more often mentioned being influenced in their sexual practices by their peers than by their families. Sexually active students reported lower self-efficacy to refrain from sex than abstainers. Interventions that seek to promote abstinence among those willing to achieve this should stress the advantages of abstinence from sex until marriage, offer tools to resist peer pressure and enhance self-efficacy to abstain. These findings can be used to develop comprehensive HIV prevention programmes that primarily promote abstinence among university students who are not yet sexually active but also consider promoting condom use and other safer-sex practices among those who are sexually active. These interventions should also be gender-sensitive to address the needs of both male and female students.

## Introduction

Sudan is one of the largest Islamic countries in Africa. Its population is about 34 million, of which about half are between 10 and 35 years of age with almost equal gender distribution (Sudan National AIDS Program [SNAP], 2014). Following the separation of South Sudan, it has been estimated that about 95% of the population are Muslims. A survey conducted in 2002 has shown that Khartoum state had the highest HIV prevalence in Sudan. According to that survey, the prevalence among university students was 1.1%, raising concerns about the risk of HIV spreading among this population group (SNAP, 2002). A previous survey has shown a rise in pre-marital sexual practices among university students in Sudan; from 6.5 to 12.5% between 2002 and 2010 while condom use is still very low among this group (32.4% during last sexual intercourse) (SNAP, 2010). These changes have also been observed in comparable Arab and Islamic communities (Massad et al., 2014; Raheel, Mahmood, & BinSaeed, 2013). HIV is still a public health problem in Sudan. In 2016, the estimated number of new HIV infections in Sudan was 5000 [1900–9400]. It was also estimated that 56 000 [34,000–87,000] people were living with HIV in Sudan in the same year (UNAIDS, 2017).

Condom promotion programmes as an effective HIV prevention strategy are difficult to implement in many Islamic countries where all types of extramarital sex are forbidden. It has been tried in some Islamic countries to promote condom use by enforcing messages about the importance of health and the preservation of human life in Islam. However, this issue still causes a significant tension and lack of trust between health policymakers and religious leaders who believe that condom promotion will promote immorality and promiscuity (Barmania & Aljunid, 2016; Kamarulzaman, 2013). In Islamic communities, social norms expect sexual abstinence; virginity at the time of marriage remains a virtue, while sex outside marriage is considered sinful (Zain Al-Dien, 2010). However, recent studies among university students in some Islamic countries have shown that many students engage in pre-marital sex. Moreover, the majority of sexually active students reported having multiple sexual partners and inconsistent condom use. They also reported a very low level of HIV/STI risk perception. Watching pornography has been observed as a common predictor of sexual behviour among this population (Khalajabadi Farahani, Akhondi, Shirzad, & Azin, 2018; Raheel et al., 2013). As explored in a previous qualitative study, youth in Islamic communities may engage in pre-marital sex for several reasons including personal pleasure, challenging the culture, proving manhood, facing financial constraints and inability to marry (Massad et al., 2014).

A recent meta-analysis of 63 trials conducted in non-Islamic communities to reduce the risk of sexually transmitted infections in adolescents concluded that the interventions which did not promote abstinence were more successful in promoting condom use (Morales et al., 2018). Another meta-analysis that included 67 studies mostly in the United States also found that the interventions which focused on abstinence as a goal were less successful in reducing the sexual risk of HIV (Johnson, Scott-Sheldon, Huedo-Medina, & Carey, 2011). However, it could be argued that these results may not be generalisable to Islamic communities where abstinence from sex until marriage is the norm. Besides, previous research has shown that the interventions which incorporate a community's social norm are more likely to be accepted and more extensively implemented (Marston & King, 2006; Willems, 2009). Despite these arguments, the findings of these meta-analysis studies highlight the importance of adopting a more comprehensive approach to HIV prevention. Therefore, comprehensive HIV prevention programmes that aim to promote sexual abstinence among those who are not yet sexually active in addition to promoting condom use and other safer-sex practices among the sexually active could be a useful strategy to reduce HIV infection in Islamic communities, such as Khartoum. Some of these interventions have positively impacted on both short and long-term safe sex practices and abstinence (Aarons et al., 2000; Jemmott, Jemmott, & Fong, 1998; O'Donnell et al., 2002; Underhill, Operario, & Montgomery, 2007). A systematic review of such interventions has affirmed that this approach has no undermining effect on safe sex or abstinence messages (Underhill et al., 2007). Comprehensive HIV programmes are also more likely to be accepted among religious leaders than purely harm reduction strategies and could also play an essential role in building trust and encouraging religious leaders to participate in HIV control (Trintapoli, 2011). However, to be effective, such interventions need to address, amongst other things, the most salient beliefs students have relating to sexual abstinence. So far, that information is not available for university students in Sudan.

The main objective of this study was therefore to explore sexual abstinence behaviour, sexual practices and pre-motivational, motivational and post-motivational beliefs about abstinence among university students in Khartoum. Since, to the authors` knowledge, sexual behaviour and beliefs regarding voluntary abstinence have not been studied in-depth within this population before, a qualitative design was used (Power, 2002). The I-Change Model was used as a theoretical framework for this purpose.

**Figure 1.**
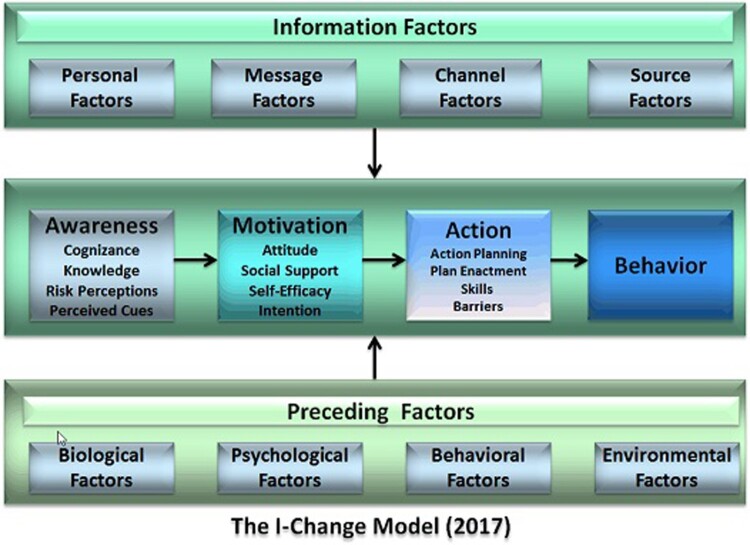
The integrated behavioural change (I-Change) model.

The Integrated Model for Change (I-Change Model), (Figure 1) integrates several social cognitive theories including the Theory of Planned Behaviour, The Health Belief Model and Socio-Cognitive Theory (Eggers et al., 2017) and has been successful in predicting health-related behaviours, including sexual health behaviours (de Vries et al., 2014; Dlamini et al., 2009; Eggers et al., 2016; Huver, Engels, & de Vries, 2006). The I-Change Model distinguishes three phases: a pre-motivational, motivational and post-motivational phase (de Vries et al., 2003). The pre-motivational phase is the awareness phase in which individuals become aware of a problem and their own risks. Awareness is determined by knowledge, risk perceptions, cues to action and cognisance about their own behaviour. If awareness about a health problem and its risk behaviours is developed, individuals can proceed to the motivational phase in which they will consider taking up a health-promoting behaviour or reducing their risk behaviour. A person's motivation or intention to do this is determined by attitudes, social influence and self-efficacy (de Vries, Mesters, Steeg, & Honing, 2005; de Vries, 2017). A person's attitude consists of the perceived cognitive and emotional advantages and disadvantages of the behaviour (de Vries et al., 2005). Social influence perceptions are determined by the perception of others carrying out a specific type of behaviour (social modelling), the social norms and the social support to adopt the behaviour (de Vries et al., 1994). Self-efficacy refers to a person's perception of their capability to carry out a type of behaviour in a variety of situations (de Vries, Dijkstra, & Kuhlman, 1988). Together, these motivational factors predict the intention to adopt certain healthy behaviour. The translation of intention into behaviour is the third and post-motivational phase which is determined by a person's level of intention, self-efficacy, action planning, plan enactment and the level of barriers that are encountered (Eggers et al., 2017).

## Materials and methods

### Design

For the purpose of this study, a qualitative study design was used and 30 semi-structured individual interviews were conducted. This study adheres to the Qualitative Research Review guidelines (RATS) (Clark, 2003).

#### Recruitment and participant selection

A purposive sample of 15 university students from five non-religious universities (Three public and two private) and with different characteristics was initially invited to participate in the study. This sample included both male and female students with varied sexual histories (including sexual abstinence), socioeconomic statuses, backgrounds (rural or urban), fields of study, current academic year and university type attendance. Additional participants were recruited by the already selected students (i.e. snowball recruitment); interviewing continued until data saturation was reached (Kitzinger, 1995).

### Data collection

#### Measurement

An interview guide was designed by the research team to refer to during the interviews. As the Integrated Change Model served as the framework, a deductive approach was used to guide the development of the semi-structured interview questions (Elo & Kyngäs, 2008). The questions were designed to explore abstinence, sexual practices and their pre-motivational, motivational and post-motivational determinants, including knowledge, risk perception, attitude, social influence and self-efficacy. Table 1 below summarises the interview guide. A pilot study was performed with five university students other than those who participated in the main study. Based on their feedback, minor linguistic modifications to the interview guide were made such as using the term ‘abstinence from sex until marriage’ instead of ‘abstinence from sex’ and using ‘pre-marital/ extramarital sex’ instead of ‘sex’.

**Table 1.** Summary of study interview guide.

A. General personal and demographic data	Age	
	Gender	
	Marital status	
	Type of university	
	Field of study	
	Family residence	
	Sexual behaviour (abstainer or sexually active)	
B.1. Exploring the participant’s behaviours and beliefs about premarital and extramarital sex.	Introduction	B.1.1. What do you think about the premarital and extramarital sexual practices among university students?
	Knowledge	B.1.2. What do you know about HIV and its transmission and prevention
	Risk perception	B.1.3. What are/could be the risks that can be associated with these sexual behaviours?
	Attitude	B.1.4 What are/could be the disadvantages of engaging in premarital/extramarital sex?
		B.1.5 What could be potential advantages of engaging in premarital/extramarital sex?
	Social influence	B.1.6 Which people would support you to practice premarital/extramarital sex?
		B.1.7 Who would be against you practising premarital/extramarital sex?
	Self-efficacy	B.1.8 When will it be difficult for you to resist being engaged in premarital/extramarital sex?
	Other factors	B.1.9 What are the other factors which encourage you to be engaged in premarital sex?
B.2. Exploring participant’s behaviours and beliefs about abstinence from sex.	Introduction	B.2.1. What do you think about abstinence from sex until marriage?
	Attitude	B.2.2 What are/could be the advantages of abstaining from sex until marriage?
		B.2.3 What are/could be for you the disadvantages of abstaining from sex until marriage?
		B.2.4. What role if any could abstinence from sex until marriage play to protect you from getting HIV?
	Social influence	B.2.5 Who would support you to remain abstinent from sex until marriage?
		B.2.6 Who would be against you remaining abstinent from sex until marriage?
	Self-efficacy	B.2.7 When is/would it be difficult for you to remain abstinent from sex until marriage?
	Other factors	B.2.8 What are the other factors that encourage you to remain abstinent from sex until marriage?

#### Procedure

The individual interviews were conducted in August 2014 by the principal researcher and two well-trained HIV counsellors (a male and a female) with experience in conducting qualitative interviews. Individual interviews were held at the private counselling rooms of the Voluntary Counselling and Testing (VCT) centres inside their universities or the students` hostels. Each participant was interviewed separately and only the participant and the interviewer were present during each interview. Each interview lasted between 50–75 min. Each participant was asked to have the interview audio recorded. All male participants agreed, while most female participants refused for privacy concerns. For the ten female students who refused audio recording their interviews, detailed written notes were taken by their interviewers during the interviews and these notes were then included in the analysis. The interviews were carried in Arabic and all the audio-recorded interviews were transcribed verbatim and revised by the interviewers before starting data analysis.

### Data analysis

Transcribed interview data and written notes were revised and validated by the researcher and his assistants. Based on the interview questions and the collected data, an initial coding scheme was designed and agreed upon by the research team. Additional sub-codes were added to the coding scheme during the coding procedure. Data were analysed using Nvivo version 10. Data were interpreted and themes were integrated and linked to obtain a rich and deep understanding of the data collected (Bazeley, 2009). The important themes identified were supported by quotes from the participants` interviews.

#### Ethical consideration

The procedures followed were in accordance with the ethical standards of the institutional and national research committee and with the Helsinki declaration of 1975, as revised in 2008. Ethical approval was obtained from the Directorate of Research, Ministry of Health, Khartoum State in July 2014. All participants were informed about the objectives of the study and the confidentiality of their data. To protect their privacy, all identifying information was deleted from the data and codes were used instead. They were also informed that participation is voluntary. Informed consent was then obtained from all individual participants included in the study.

## Results

To check the validity of the coding process, two interviews were fully coded by the principal researcher and an assistant, as well as all interviews for a selection of codes (n=5). The inter-coder variation was then calculated using both the percentage of agreement and Kappa. The results showed an agreement percentage of 98.8 (92.6-100) and Kappa of 0.88 (0.61- 0.98), indicating good inter-coder reliability.

### Characteristics of the participants

Thirty university students were interviewed in this study. The sample included 16 male and 14 female students studying at six different universities in Khartoum state (Sudan). All of the participants were Muslims and their age ranged from 18 to 24 years (M=19). Eleven respondents (six males and five females) reported being currently sexually active. Table 2 below summarises the participants’ characteristics.

**Table 2.** Characteristics of the study participants.

		Sexually active			Abstainers		
		Male	Female	Total	Male	Female	Total
Total number of participants		6 (20 %)	5 (17 %)	11 (37 %)	10 (33 %)	9 (30 %)	19 (63 %)
Age group	18 -20 years	2 (7 %)	2 (7 %)	4 (13 %)	5 (17 %)	4 (13 %)	9 (30 %)
	21–24 years	4 (13 %)	3 (10 %)	7 (23 %)	5 (17 %)	5 (17 %)	10 (33 %)
Residence	Khartoum state	4 (13 %)	3 (10 %)	7 (23 %)	7 (23 %)	6 (20 %)	13 (43 %)
	Other states	2 (7 %)	2 (7 %)	4 (13 %)	3 (10 %)	3 (10 %)	6 (20 %)
Type of university	Public	3 (10 %)	3 (10 %)	6 (20 %)	3 (10 %)	3 (10 %)	6 (20 %)
	Private	3 (10 %)	2 (7 %)	5 (17 %)	7 (23 %)	6 (20 %)	13 (43 %)
Field of study	Medical field	2 (7 %)	2 (7 %)	4 (13 %)	6 (20 %)	5 (17 %)	11 (37 %)
	Art & Education	4 (13 %)	3 (10 %)	7 (23 %)	4 (13 %)	4 (13 %)	8 (27 %)

### Sexual practices among university students

Most of the sexually active participants, including both male and female students, indicated that they had heterosexual relations with multiple sex partners. Sexual relations among university students were mostly incidental and not part of steady relationships as one of them explained, ‘*Most of my sexual relations were accidental and temporal and only for lust’* (23-years-old male, sexually active). Most of the participants also declared practising sex without condoms. One of them said, ‘*only one of the girls with whom I had sex asked me to use a condom and I had one at that time. The rest of the girls were not much concerned about that and did not mind having sex without a condom’* (21-year-old male, sexually active).

Both male and female abstainers considered abstinence the behaviour which everybody needs to adopt. As seen by a female participant, abstinence is ‘*a basic principle as it preserves our dignity and position in the community’* (18-year-old female, abstainer).

### Knowledge

Most of the male and female abstainers and sexually active participants had some knowledge about HIV and the way is it transmitted. Unprotected pre-marital sex, multiple partners and anal sex were all identified as risky sexual behaviours. Despite this knowledge, More than half of the male abstainers reported clear misconceptions about HIV infection and its transmission. One of them believed that ‘*It is very dangerous to have infected people in the community because the virus can be transmitted through saliva and using common drinking cups*’ (24-year-old male, abstainer). Besides, the sexually active male participants had misconceptions about condom use and its protective role against HIV. Some of them had never seen condoms before, and the majority lacked knowledge on how to use condoms. Generally, It was observed that female participants had higher knowledge about HIV than male participants.

### HIV risk perception

No difference in the perception of susceptibility to HIV was observed between the abstainers and the sexually active or between the male and female participants. All of them perceived the risk of getting HIV if they practised sex as high. They all believed that being university students put them at higher risk because they were ‘*'more likely to practice sex with many partners and it is difficult to know if these partners have AIDS or not*’ (21-year-old male, sexually active). Although all of the participants also believed that HIV is a serious problem, female abstainers seemed to have a higher perception of severity than the other participants. One of them described HIV as the most serious disease and another called it the plaque of the century.

### Cues to action

Different types of cues were mentioned by both male and female students. These cues were believed to influence their tendency to remain abstinent or become sexually active. Some female abstainers expressed how they were encouraged to abstain after hearing HIV infected persons tell their own stories and experiences of living with HIV. One of them said ‘*one day; I saw an infected person inside my university. He was talking about his story. I felt very sorry for him as he was young. I started to think about his family and what they did when they found out that he had HIV. Then I decided never to practice sex before marriage*’ (22-year-old female, abstainer). Some of the abstainers also pointed to the rule of some religious cues such as listening to spiritual advice about abstinence, reading the Holy Quran and praying, which they considered important cues to abstinence.

On the other side, the male and female students who were sexually active also expressed different cues that encouraged them to practice sex. Some sexually active females admitted that the urgent need for money was one of the most important cues to practice sex. Among the sexually active male students, girls` seduction and watching internet pornographic movies were considered the main cues to sexual practices, ‘*the girls in our university wear very attractive clothes, which raises our lust. We cannot control ourselves, especially when we watch the pornographic videos exchanged through Whatsapp and Facebook*’ (23-year-old male, sexually active).

### Attitude towards abstinence and sexual practices

Both male and female participants, including those who are sexually active, had a positive attitude towards abstinence. Almost all of them believed in the religious, social, psychological, physical and academic advantages of abstaining from sex until marriage. Expressing his positive attitude towards abstinence, one of the female abstainers said, ‘*Abstinence from sex helps me to concentrate on my university study. It makes me more close to Allah because these practices are forbidden in our religion and Allah will not be satisfied with us. By abstaining, I preserve my dignity and my family's reputation. I also protect myself against diseases’* (24-year-old female, abstainer). Another sexually active male student compared between abstinence and sexual practices saying, ‘*I do not think that by abstaining and avoiding sexual relations I will lose anything because believing in abstinence and adhering to this belief is the best feeling. This is a feeling which is much greater than sexual joy and pleasure. I regret that I did not maintain my chastity’* (24-year-old male, sexually active).

Both male and female participants including the sexually active students expressed a negative attitude towards pre-marital and extramarital sex. They indicated that these sexual relations are religiously forbidden sins and described them as impolite, unacceptable and immoral behaviours. ‘*Such things should never happen; I wonder how they do that. Students should not do such wrong deeds’* (24-year-old female, abstainer), ‘*In my opinion, if one fails to control himself when having sexual desire or lust, there will be no difference between him and the animals. However, sometimes it happens to me that I follow my desires and forget the consequences’* (24-year-old male, sexually active).

Despite this negative attitude, sexually active male and female participants shared some perceived advantages of pre-marital sex, such as responding to the natural drives and enjoying sexual pleasure. However, each group also had their own perceived advantages. The male students disclosed that they practised sex to prove masculinity, adulthood and sexual ability to their peers. They also claimed that having sex would prevent the harmful effects of semen stagnation. Additionally, some male students believed that practising sex during exam times could alleviate academic stress. On the other hand, some female participants pointed to the financial benefits of having sexual practices. However, most of these perceived advantages of sexual practices were deemed immediate but temporal and were usually followed by negative emotions such as regret, ‘*It is only temporal pleasure and satisfaction that lasts for one or two hours to leave you with sorrow and regret when you start to think about the consequences*’ (21-year-old male, sexually active). This was observed among both male and female students.

Regarding the perceived disadvantages of pre-marital sex, the sexually active participants, regardless of their gender, described an association between their sexual practices and mental distress and social stigma: ‘*the worst thing is the psychological pain of guilt and regret in addition to the continuous thinking about my reaction if one day I find one of my sisters in a similar condition. This fear and stress last for months. I start to cry and cry as I have hundreds and hundreds of questions for which I do not have answers. I feel remorse and become unable to do my daily activities. Even if I do anything, I do it without interest*’ (21-year-old male, sexually active). They also talked about physical illnesses, legal consequences and poor academic performance as unfavourable results of their sexual practices.

Socially, the participants believed that sexual practices could spread immoralities, spoil the youth and increase the number of illegal children. Pre-marital sex was also considered a gateway to alcohol and drug addiction. The abstainers and sexually active participants of both gender discussed the association between pre-marital sex and the social stigma. However, female participants were more concerned about its profound negative impact on family reputations, ‘*I know that many people will criticise me if they know that I am practising sex. My family will be affected, as well. Any man who decides to be engaged with one of my sisters or with me if he knows that I have sexual relations; indeed, he will change his mind’* (21-year-old female, sexually active). Pre-marital sexual practices were also thought to be associated with criminal problems such as rape crimes, suicidal attempts and illegal abortions.

Physically, both the abstainers and sexually active students talked about the association between pre-marital sexual practices and HIV and other sexually transmitted infections. One of the male abstainers asserted that pre-marital sex could cause impotence and infertility.

Academically, some participants described how some girls were prevented from completing their university study fearing the shame that might be brought to their families because of their sexual practices. Pre-marital sexual practices also affected some university students` academic performance as declared by a participant, ‘*Students become very busy with sex. One of my colleagues, whenever I sit with him, he starts to talk about his sexual adventures. He can spend all day talking about sex’* (24-year-old male, abstainer). Most of the expressed disadvantages were universally believed to affect female students more than the males due to the social norms and traditions which look at female virginity as something of great value not only for the girl but also for her family, tribe, society and even her future offspring.

### Social influence on sexual behaviour

The study participants considered the university community as a major driver for sexual practices among the students. However, both male and female abstainers pointed to the role of their family members, especially parents, who were believed to be the main supporters of abstinence. Their influence was indirect in most of the cases, due to the social norms which prohibited talking openly about sexual issues between family members as participants mentioned. Female abstainers perceived greater family influence on their sexual behaviour since their engagement in sexual practices could affect the whole family reputation and bring shame to them much more than their male partner. This was indicated by one of them who said, ‘*When I was accepted to join the university, my family talked to me about the university community and the different groups of people there with both good and bad manners. They advised me to select my friends carefully. They also told me to be very serious with any boy who alluded to [sex]’* (18-year-old female, abstainer).

On the other side, the sexually active students, especially male students, declared that they were more influenced by their peers than by their families as one of them admitted; ‘*I am not too close to my father to talk to him frankly about sex but my friends are closer because we are at a similar age. I tell my close friend about many things that happen to me while I cannot do that with my dad or mom. My peers are always persuasive’* (18-year-old male, sexually active). Seniors and sexually active peers were believed to be the main supporters who encouraged new male and female students to practice sex as one of the female participants mentioned, ‘ *‘my university friends encouraged me a lot to practice sex. We are very much affected by our senior colleagues whom we consider our models’* (19-year-old female, sexually active). One of the male participants also described how he was pushed by his peers to practice sex, saying, ‘One day, *I was invited by some girls at the university to have sex. When I refused, they started to talk about me with my friends. They questioned my manhood’* (21-year-old male, sexually active). Another one said ‘*I always listen to my friends talking about sex and the different ways of doing it: vaginal, anal and oral sex. Listening to these things makes me very eager to practice it’* (23-year-old male, sexually active). Both male and female students also pointed to the role of media and internet websites in promoting sexual practices among students. The pornographic movies could be easily accessed and exchanged between students through their smartphones as one of them expressed, ‘*Many times before, I was about to practice sex. The internet has a great effect. It is one of the most encouraging causes because we can stay hidden from peoples` eyes and search porn websites, which raise our lust. It is the easiest way to go astray’* (20-year-old male, abstainer)

Of equal importance, the participants, regardless of their gender or sexual behaviour, pointed to the role of religion and religiosity. All of them believed that religious principles act against pre-marital sexual behaviours. However, male abstainers more often mentioned to be directly influenced by the religious scholars at mosques who support abstinence and discourage all forms of religiously forbidden sexual relations. One of them said, ‘*The Imam of our mosque gives special care to spiritual and faithful education. He always talks about extramarital sex as a religiously forbidden and immoral behaviour*’ (20-year-old male, abstainer). Religion, as implied by some participants, not only supports abstainers to remain so but also makes some sexually active students contemplate changing their sexual behaviour and become abstainers. This role of religion was expressed by one of the sexually active students who said, ‘*Pre-marital sex is a sin which Allah warned us not to commit it. I know this very well but sometimes I fail to overcome my desires and I forget. Then I remember again and feel sorrow and regret. The last time I had sex, I listened to Azan [i.e. call for prayers] but did not reply to it because I was busy with sex. When I finished, I felt pangs of remorse and swore never to practice [pre-marital] sex again’* (21-year-old male, sexually active).

### Self-efficacy

Compared with the sexually active participants, abstainers, especially females, seemed to have higher self-efficacy to remain abstinent from sex until marriage. One of the female students expressed her confidence in her ability to stay abstinent and said, ‘*Inside my university, I am so cautious in my relations. I avoid anything that may lead me to practice these behaviours from the start. This is why I have never faced such things’* (19-years-old female, abstainer). Another one added, ‘*I do not think that I face any difficulty in remaining abstinent as long as I am fully alert’* (19-year-old female, abstainer).

Conversely, sexually active male and female participants perceived themselves as lacking the capability to resist the problematic situations that encourage them to practice sex as one of them disclosed, ‘*To stop having sex is something challenging. Even if I manage to abstain for one month or two, I cannot abstain until marriage. Many times before, I decided to abstain but I failed’* (23-year-old male, sexually active). This lack of confidence was also affirmed by another participant who declared, *‘I find it difficult to resist my desires when a partner shows me the attractive parts of his body and arranges a safe place for sex. I have become so addicted to it that I no longer care about the dangers of these relations and the diseases they may cause’* (21-year-old female, sexually active).

The difficult situations affecting both male and female students’ self-efficacy to abstain from sex until marriage included watching sexual and pornographic movies, practising masturbation and consuming alcohol and drugs. Having free time and being alone with the partner at home or in isolated hidden places were also believed to increase the chance of engaging in sexual activity. The majority of the male students also described how they were seduced by girls uncovering their hair, exposing some parts of their bodies, using perfume or kissing and hugging each other in front of them. One of them said, ‘*every day we face exciting things in the university community that push us to do these things. Everybody knows that girls have become very attractive nowadays by the way they dress, walk and talk. We cannot resist their attraction’* (23-year-old male, sexually active). On the other side, the need for money was identified as one of the challenges that mainly affected some female students’ ability to abstain. One of the sexually active girls described how she felt it difficult not to have sex whenever she had an urgent need for money. She said, ‘*I am convinced that I should abstain from pre-marital sex but I always hesitate and return to sex whenever I need money’* (24-years-old female, sexually active).

### Action plans

In their attempts to remain abstinent from sex until marriage, male and female abstainers relied on adherence to religious values, not watching pornographic movies and avoiding potential sexual partners. When asked about his plans to remain abstinent, a male participant replied, ‘*Whenever these ideas come to me, I immediately get rid of it by reading the Holy Quran and religious books. Sometimes I go to my Sheikh to ask him. I know that the problem is in having free time. The more free time we have, the more Satan will lead us astray’* (24-year-old male, abstainer). Additionally, some male abstainers talked about filling their free times practising hobbies to avoid thinking about sex.

Female abstainers also had their own action plans such as avoiding social relations with male students and not sharing *Whatsapp* and *Facebook* groups with them. Moreover, seeking financial support from some charity organisations assisted some of the sexually active girls to abstain as one of them expressed, ‘*Recently, I have started to abstain from sex. I have been much encouraged by the health education programme of an organisation as well as the income-generating project they provided me with’* (24-year-old female, sexually active).

## Discussion

The main objective of this study was to explore sexual abstinence, sexual practices and beliefs among university students in Khartoum for which we interviewed 11 sexually active students and 19 students that were not.

Consistent with previous studies in similar Arab countries (Massad et al., 2014; Raheel et al., 2013), the study participants viewed practising (unprotected) sex as a fairly common behaviour among university students. This change in sexual behaviours after starting at the university has also been observed in several studies in different communities (Chanakira, O'Cathain, Goyder, & Freeman, 2014; Farrow & Arnold, 2003; Othero, Aduma, & Opil, 2009). Concerning awareness, some misconceptions about HIV and its transmissions were identified but no marked difference in HIV-related knowledge was observed between the abstainers and sexually active students. However, some abstainers lacked conceptual knowledge about HIV transmission, while many sexually active students lacked knowledge about how to protect themselves against HIV. Female students seem to have more comprehensive knowledge and fewer misconceptions about HIV than their male counterparts, which could be attributed to having different sources of knowledge. In contrast, a previous study conducted in Nigeria reported lower HIV knowledge among females than males (Oginni, Adebajo, & Ahonsi, 2017). Therefore, further evaluation of the gender-related difference in HIV knowledge is needed to identify the educational needs of both male and female students.

Perceptions of HIV susceptibility did not seem to be different between abstainers and sexually active students in this study as well although some previous studies identified HIV risk perception as an important predictor of risky sexual behaviour (Akwara, Madise, & Hinde, 2003; Nkomazana & Maharaj, 2014). Most of the students perceived their increased vulnerability to HIV due to their increased social freedom as university students and increased chances of engaging in sexual behaviours. No difference in the perception of HIV susceptibility was observed between male and female students. However, female students expressed a higher perception of HIV severity. The association between HIV and the forbidden sexual behaviours and the severe social consequences of disclosing females’ pre-marital sex rather than the impact of HIV on health may justify this difference in perceived seriousness of HIV. Parallel to previous research (Green & Witte, 2006; Tannenbaum et al., 2015), our findings suggested that participants` experience with people living with HIV/AIDS (PLWHA) could increase their fear of contracting HIV and thus influence their sexual behaviour. This may recommend the use of fear appeals in HIV-prevention messages and the involvement of PLWHA in HIV risk reduction interventions. In contrast, some studies suggested that fear appeals in HIV-prevention messages may not be effective in changing HIV sexual risk behaviour (Albarracin et al., 2005; Earl & Albarracín, 2007). Moreover, It has been suggested that such messages may be harmful as they may increase the stigma and discrimination against PLWHA (Bastien, 2011). However, the influence of fear appeals on sexual risk behaviours is believed to be dependent on different personal factors such as self-efficacy as well as the social context of the audients (Bastien, 2011). Therefore, more research is needed to explore the influence of such messages on this population.

Regarding students` attitude towards abstinence and pre-marital sex, the study showed that both abstainers and sexually active students had a positive attitude towards abstinence, with virginity highly valued, especially among female abstainers. Generally, there was agreement about the potential advantages and disadvantages of pre-marital sexual behaviours among both abstainers and sexually active respondents. However, sexually active students tended also to value the perceived temporary but immediate advantages of practising pre-marital sex while abstainers considered the long-term physical, mental, social, religious, academic and criminal disadvantages of these sexual practices. Looking at sex as a sign of masculinity and manhood was observed in this study as well as some previous studies (Fleming & Davis, 2018; Fleming, DiClemente, & Barrington, 2016; Massad et al., 2014) and affected many male students` attitude towards pre-marital sex. Addressing this concept within health communication and promotion interventions using discussions and arguments seems to be important (Eldredge et al., 2016; Mulugeta & Berhane, 2014). Our findings also suggest that communication strategies need to address the feelings of anticipated regret that may occur when refraining from abstinence (Eldredge et al., 2016). Moreover, to build a positive attitude towards abstinence from sex until marriage, some messages should be tailored to address the gender differences in perceived advantages of pre-marital sex.

Regarding social influence, peer pressure appeared to be very influential in both male and female students` sexual behaviours, especially among the junior students. This finding is consistent with many previous studies (Fearon, Wiggins, Pettifor, & Hargreaves, 2015; Mulugeta & Berhane, 2014; Othero et al., 2009; Tura, Alemseged, & Dejene, 2012). In our study, direct active peer pressure was observed as an important mechanism of peer influence on students` sexual behaviour. The role of peers in young people sexual behaviours is poorly studied in this community but several mechanisms have been identified through research in other communities. These include the perceived peers` norms favouring sex, affiliation with antisocial peers, providing opportunities for meeting potential sexual partners and active peer pressure (Capaldi, Stoolmiller, Clark, & Owen, 2002; Cavanagh, 2004; Van de Bongardt, Reitz, Sandfort, & Dekovic, 2015). Therefore, more investigation is needed to explore the role of the other mechanisms of peer pressure and identify differences between male and female students in this regard. This exploration is fundamental to design effective, tailored interventions to mitigate peer influence (Bingenheimer, Asante, & Ahiadeke, 2015). Also, to alleviate peer pressure, students need to be psychologically immunised against pressure and equipped with some skills to resist it (Eldredge et al., 2016).

On the other hand, our study pointed to the role of parents and other family members in supporting abstinence, especially among female students. Our findings also paralleled some previous studies and suggested that the imbalance between moderate family influence and high peer pressure could have a substantial impact on student's sexual practices (Legesse, 2012; Tan & Gun, 2018). Although the involvement of parents and family members in abstinence-promoting interventions may be beneficial to mitigate peer influence, the feasibility of family involvement in such programs may be questioned. Therefore, reliance on social inoculation and peer resistance methods (Compton, Jackson, & Dimmock, 2016) may be more relevant for university students; methods also tried and shown to be effective in other countries (Parker, Ivanov, & Compton, 2012).

In addition, the study identified the role of religious leaders, as very influential and trustworthy role models, in supporting abstinence until marriage, a conclusion also shared with some previous studies (Abu-Moghli, Nabolsi, Khalaf, & Suliman, 2010; Trintapoli, 2011). Religious leaders can be deeply involved, trained and encouraged to participate in HIV control and abstinence-promoting interventions (Abu-Moghli et al., 2010; Ucheaga & Hartwig, 2010). Their participation may enrich such programmes with religious messages to build a positive attitude towards abstinence and enhance self-efficacy to abstain from sex until marriage. Their involvement in designing and implementing such programmes could also increase their commitment to HIV prevention and facilitate their use of a common language with public health workers, which will, in turn, create an enabling environment to implement other HIV harm reduction strategies (Barmania & Aljunid, 2016; Trintapoli, 2011).

Concerning self-efficacy and in line with some previous studies, our findings identified self-efficacy as an important determinant of sexual behaviour among university students as well (Taffa, Klepp, Sundby, & Bjune, 2002; Viseskul, Fongkaew, Settheekul, & Grimes, 2015). We observed a higher level of self-efficacy to remain abstinent among female students when compared to male students. This observation could explain why females tended to remain abstinent while more male students engaged in sexual practices. Several factors affecting students` self-efficacy to abstain from sex until marriage, both positively and negatively, were uncovered. Male and female students appeared to be challenged by different problematic situations that reduced their self-efficacy to abstain. Seduction by girls, watching pornographic videos and alcohol affected male students` ability to refrain from pre-marital sex.

On the other hand, the urgent need for money seemed to have a more significant effect on female students’ sexual behaviours as observed in a similar previous study. However, it could be argued that some female students talk about their need for money only to justify their sexual behaviour. Therefore, it is recommended to include socioeconomic evaluation in future research to explore the association between poverty and female students` sexual behaviours, including both commercial and transactional sex (Longo et al., 2017).

Realising strong self-efficacy to cope with such situations is vital in order to provide students with sufficient confidence to be able to refrain from pre-marital sex. Considering the diversity of factors influencing their self-efficacy to abstain, male and female students should be prompted to list their own barriers, plan their individual coping responses to overcome these barriers and practice these coping responses. Self-monitoring of the behaviour could also be useful where student are encouraged to keep a record of their sexual behaviour to find out why and when they fail to abstain. Cue altering, changing a stimulus that elicits the behaviour, is also one of the successful methods of overcoming barriers. This method was also mentioned by some of the participants who described how they changed sexual stimuli by reading the holy Quran or practising their hobbies.

## Practice implications

Despite the argument against abstinence promotion programmes in many countries, this study highlights the importance of these programmes in Islamic communities like Sudan where abstinence from sex until marriage is the norm; sexual practices are commonly followed by social, psychological and legal consequences and condom promotion alone is not yet a feasible and socially acceptable approach to reduce HIV infections. However, to prevent HIV among university students in Sudan, promotion programmes with a more comprehensive approach (including both voluntary abstinence and safe sex practices) are needed. Such programmes are required to address the different psychosocial determinants identified in this study. Of equal importance and considering the differences in the psychosocial determinants of abstinences and sexual practices between male and female students, these interventions should also be gender-sensitive. Misconceptions about HIV and its transmission must be addressed and risk perception could be raised by encouraging the students and helping them to perform personal risk assessment properly. To build a positive attitude towards abstinence, the promotion programme should stress the advantages of abstinence from sex until marriage and help the university students to give higher value to the long-term advantages of abstinence as compared to the temporary advantages of sexual practices. This could be achieved through different behavioural change techniques such as arguments, self-reevaluation and the anticipated regret method (Brewer, DeFrank, & Gilkey, 2016). As revealed by the study, addressing peer influence should be an essential component of the programme as well. The programme could include training in pressure resistance skills as well as social inoculation methods to mitigate peer pressure. Considering the importance of self-efficacy as a determinant of sexual behaviours among this population, the promotion programme should also aim at enhancing students` confidence and ability to overcome the observed abstinence barriers. Suggested self-efficacy enhancement strategies include verbal persuasion, self-monitoring of behaviour, planning coping responses and cue altering. Alternatively, and for those who do not intend to abstain from sex until marriage, stimulation of condom use, for instance, should be addressed through the promotion programme as well. It also seems to be essential to involve religious leaders, teachers, nurses and other health professionals in delivering such programmes to fill the huge gap in sexual health education in schools curriculum.

## Strengths and limitations

This is the first study that explores the sensitive issues around sexual behaviours among both male and female university students in Sudan in-depth. The participants felt very much at ease with the interviewers because of the precautions that were taken to protect their identity. Yet, the sample only consisted of 30 university students. Thus, it may not be representative of the Sudanese student population. As the study was qualitative, no statistical inferences can be made regarding the most significant factors. Hence, a follow-up study using a longitudinal quantitative approach is recommended.

## Conclusion

HIV prevention among university students in Sudan requires comprehensive programmes that promote both abstinence from sex until marriage and condom use and other safe sex practices. To promote abstinence from sexual practice until marriage, which is in accordance with the Quran and Islamic values, these programmes should stress the advantages of abstaining from pre-marital sex, offer tools to resist peer pressure and enhance self-efficacy to abstain. At the same time, programmes for students who are unwilling or perceiving themselves as incapable of voluntary abstinence may also be needed in order to prevent unwanted pregnancies and sexually transmitted diseases, including HIV. Research is required, however, to investigate how such programmes implementation could be facilitated by involving religious leaders and the Islamic values of compassion and prevention of harm and disease.
